# Ethical perspectives in palliative care for chronic patients: a systematic review of nurses’ experiences in home and hospital settings

**DOI:** 10.1186/s12904-026-02032-0

**Published:** 2026-03-12

**Authors:** Zahra Khalilzadeh-Farsangi, Mina Zamanifard, Fereshteh Ghasemi, Samaneh Fallah-Karimi

**Affiliations:** 1 Department of Nursing, Faculty of Nursing and Midwifery, Zarand School of Nursing, Kerman University of Medical Sciences, Kerman, Iran; 2 Department of Nursing, School of Nursing and Midwifery, Jahrom University of Medical Sciences, Jahrom, Iran; 3 Department of Community Health Nursing, Community Nursing Research Center, School of Nursing and Midwifery, Zahedan University of Medical Sciences, Zahedan, Iran; 4 Department of Midwifery Nursing, School of Nursing and Midwifery, Torbat Heydariyeh University of Medical Sciences, Torbat Heydariyeh, Iran

**Keywords:** Chronic Disease, Decision Making, End-of-Life Care, Ethical Issues, Moral Distress, Nursing, Palliative Care

## Abstract

**Background & Aim:**

The global rise of chronic diseases requiring long-term management highlights the need for effective palliative care. Nurses play a central role in providing this care, yet frequently face ethical challenges such as moral distress, complex decision-making, and occupational stress. This systematic review aimed to identify, categorize, and synthesize evidence on nurses’ ethical perspectives, challenges, and professional interventions in palliative care for patients with chronic diseases in both hospital and home settings. The review addressed the following questions: (1) What are nurses’ ethical perspectives in delivering palliative care ? (2) What ethical challenges and decision-making dilemmas do they encounter? (3) Which strategies and interventions support ethically sound care?

**Methods:**

Following PRISMA 2020 guidelines, five databases (PubMed, Scopus, Web of Science, SID, and Magiran) and gray literature via Google Scholar were systematically searched for studies published between 2015 and 2025 in English or Persian. Eligible studies included qualitative, quantitative, mixed-methods, and peer-reviewed reviews addressing nurses’ ethical challenges in palliative care. Qualitative data were analyzed using inductive content analysis, quantitative findings were synthesized descriptively, and mixed-methods results were integrated to allow convergence across study designs. Extracted codes were organized into themes, grouped into categories, and synthesized into overarching domains.

**Results:**

Thirty-four studies met inclusion criteria. Four main domains of ethical challenges emerged: (1) Clinical decision-making and patient autonomy—dilemmas regarding life-sustaining treatments, medication and fluid management, palliative sedation, and conflicts between patient preferences and family/cultural expectations; (2) Justice and resource allocation—insufficient ethics education, high workload, limited organizational support, poor interprofessional collaboration, and scarce resources; (3) Beneficence and patient-centered care—ethical issues related to technological innovations, informed consent, data privacy, and environmental pressures such as ICU or emergency care conditions; (4) Non-maleficence and moral distress prevention—moral distress, burnout, reduced ethical courage, and legal concerns affecting clinical decisions.

**Conclusion:**

Nurses encounter substantial ethical challenges in delivering palliative care for patients with chronic diseases. Addressing these challenges requires ethics education, structured organizational guidance, interprofessional collaboration, and context-sensitive strategies to safeguard patient dignity and reduce moral distress. These findings provide evidence for policymakers, educators, and managers to develop practical interventions and ethical frameworks across diverse care settings.

**Supplementary Information:**

The online version contains supplementary material available at 10.1186/s12904-026-02032-0.

## Introduction

Chronic diseases that require long-term and complex management are increasing globally, making the provision of effective healthcare critically important [1]. Palliative care is a key component of such care, aiming to improve patients’ quality of life and alleviate physical, psychological, social, and spiritual suffering [2]. According to the World Health Organization, “Palliative care is an approach that improves the quality of life of patients and their families facing problems associated with life-threatening illnesses, through the prevention and relief of suffering by early identification, precise assessment, and treatment of pain and other physical, psychosocial, and spiritual problems” [3].

Concept analyses describe palliative care as a comprehensive, proactive, and multidimensional approach. It emphasizes a holistic perspective, patient involvement in decision-making, family support, interdisciplinary coordination, and the integration of scientific knowledge and the art of care. Outcomes include enhanced quality of life, preservation of human dignity, and facilitation of patient adaptation to chronic illness [4–7].

Nurses play a central role in delivering palliative care, with responsibilities that include implementing interventions, assessing patient needs, coordinating with other healthcare team members, and supporting both patients and their families [8]. Evidence indicates that nurses caring for patients with chronic diseases frequently encounter ethical challenges, face difficult decision-making situations, and experience occupational stress.

Several studies have highlighted these experiences. Arab et al. (2022) reported that home-care nurses face conflicts between patient autonomy and family involvement, challenges in maintaining privacy, and difficulties managing cultural and religious differences [9]. Ibrahim et al. (2024) found that ethical issues related to pain management and patient rights can negatively affect nurses’ quality of life [10]. Friedrichsen et al. (2024) described how Swedish nurses experience moral distress and occupational stress when making difficult decisions regarding fluid management and care of dying patients [11]. Similarly, Akbarian-Rokni et al. (2023) observed that Iranian nurses providing palliative care to patients with end-stage heart failure encounter challenges such as pain management, psychological stress, and communication difficulties with families [12].

Nurses’ ethical perspectives encompass decisions regarding continuation or withdrawal of invasive treatments, maintaining patient autonomy and independence, safeguarding privacy, ensuring confidentiality, and balancing the needs of patients and families [13–16]. In hospital settings, these challenges are often exacerbated by time constraints, limited resources, and clinical complexity, whereas home-care nurses must navigate ethical obligations toward both the patient and the family [17, 18]. Recent research, such as Sadeghi et al. (2021), demonstrates that nurses must simultaneously manage difficult decision-making, symptom management, and provide emotional support to families while upholding ethical principles, highlighting the need for organizational support and ongoing professional education [19].

Despite the importance of these challenges, a comprehensive and systematic understanding of nurses’ ethical perspectives in palliative care for patients with chronic diseases in both hospital and home settings is lacking. Previous studies have primarily focused on specific aspects of care and provided limited insight into ethical challenges, professional decision-making, and practical interventions, resulting in fragmented information and incomplete understanding of the full ethical dimensions of palliative care.

To address this gap, this systematic review aims to summarize existing evidence on nurses’ ethical perspectives, challenges, and professional interventions in palliative care for patients with chronic diseases. Specifically, the review seeks to answer the following research questions:

1. What ethical challenges do nurses face when providing palliative care to patients with chronic diseases in hospital and home settings?
2. How do nurses navigate these challenges regarding decision-making, professional interventions, and ethical practices?
3. What strategies and organizational supports have been recommended to facilitate ethically sound palliative care in these contexts?

## Methods

### Study design and inclusion criteria

**Fig. 1:**
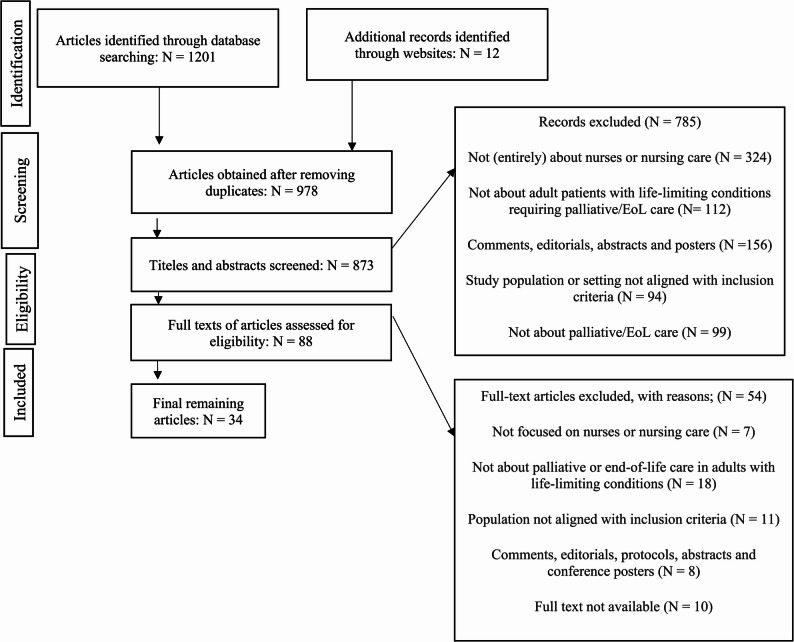
Prisma 2009 flow diagram

This study was conducted as a systematic review to identify and analyze nurses’ ethical perspectives in palliative care for patients with chronic illnesses in both hospital and home settings. The review protocol was developed following the PRISMA 2020 guidelines to ensure transparency, reproducibility, and adherence to scientific standards [20] (Fig. 1).

### Inclusion criteria

Studies were included if they met the following criteria:

1. *Population*: Nurses (registered nurses, nursing assistants, or nursing staff) providing palliative or end-of-life care to patients with chronic, progressive, or life-limiting illnesses.
2. *Focus*: Addressed ethical challenges, ethical dilemmas, moral distress, or ethical decision-making in palliative care.
3. *Study Type*: Included qualitative, quantitative, mixed-methods, quasi-experimental, experimental, or systematic and narrative reviews. Only peer-reviewed review studies were included.
4. *Keywords and Conceptual Scope*: To avoid excluding relevant studies, the conceptual scope was broadened. In addition to “ethical challenge,” terms such as “ethical dilemma,” “ethical issues,” “moral distress,” “moral conflict,” and “decision making” were included. For palliative care, terms such as “serious illness,” “life-limiting illness,” and “progressive disease” were also considered.
5. *Setting*: Conducted in hospital or home care settings.
6. *Access*: Full-text articles available in English or Persian.
7. *Time Frame*: Published between 2015 and 2025. Limiting the search to the past decade aligns with systematic review methodology recommendations [21], ensuring the inclusion of recent evidence and capturing significant changes in healthcare systems and ethical frameworks [22].

### Exclusion criteria

Studies were excluded if they:

- Were non-empirical reviews, letters to the editor, conference papers, posters, or reports without empirical data.
- Did not specifically address ethical aspects of palliative or end-of-life care.
- Focused primarily on nursing performance rather than ethical issues.
- Lacked full text.
- Focused solely on legal, policy, or administrative aspects without direct relevance to ethical issues in nursing practice.

### Search strategy

Databases searched from 2015 to April 2025 included PubMed, Scopus, Web of Science, CINAHL (via EBSCO), SID, and Magiran. Additionally, websites were reviewed to identify grey literature (Table 1).

**Table 1:** Distribution of included studies by database and study design

Database	Number of included studies	Study type (*n*)
PubMed	12	Qualitative: 6; Quantitative: 3; Mixed-methods: 1; Review: 2
Scopus	9	Qualitative: 4; Quantitative: 2; Mixed-methods: 1; Review: 2
Web of Science	5	Qualitative: 3; Quantitative: 1; Mixed-methods: 0; Review: 1
CINAHL	3	Qualitative: 2; Quantitative: 1; Mixed-methods: 0; Review: 0
SID	2	Qualitative: 2; Quantitative: 0; Mixed-methods: 0; Review: 0
Magiran	1	Qualitative: 1; Quantitative: 0; Mixed-methods: 0; Review: 0
Website / Gray literature	2	Qualitative: 0; Quantitative: 0; Mixed-methods: 0; Review: 2

The search strategy used a comprehensive set of keywords and synonyms across three main domains: (1) ethical concepts, (2) palliative care, and (3) nurses’ roles. Both MeSH terms and free-text keywords were employed. The complete PubMed search strategy is provided in Additional File 1, with search strategies for other databases also detailed therein.

References of all included studies and relevant review articles were manually screened to identify additional eligible studies. Grey literature and websites of organizations related to palliative care or nursing were also searched using relevant keywords (e.g., nursing associations, palliative care organizations, and nursing ethics committees). Duplicate articles were removed using EndNote version 26.

### Study selection and data extraction

All retrieved records were independently screened by two researchers (ZKHF and MZ) using Covidence online software (a primary screening and data extraction tool) [23]. Titles and abstracts were first screened for eligibility, yielding a Cohen’s kappa of 0.90 (unweighted), indicating almost perfect agreement according to Landis & Koch [24]. Full texts of remaining studies were then assessed by the same two researchers, with a Cohen’s kappa of 0.84, also considered almost perfect [24]. Discrepancies were resolved through discussion, consensus, or consultation with FG when needed.

Extracted data included bibliographic information, study type, country, methodology, care setting, and reported ethical challenges. For the first five studies, data were independently extracted and reviewed by ZKHF and MZ; for the remaining studies, FG extracted the data and SFK verified it.

### Quality assessment

The methodological quality of included studies was assessed using Gifford et al.‘s (2007) checklist [25], which provides different indicators for various study designs:

- *Quantitative studies*: six items including research question clarity, study design, sample selection, data collection methods, data analysis, and reporting of results.
- *Qualitative studies*: eleven items covering research question clarity, appropriateness of qualitative approach, participant selection, data collection and analysis, transparency, result validity, ethical considerations, limitations, applicability, and reporting consistency.
- *Quasi-experimental studies*: eight items including study design, participant allocation, control of confounding variables, data collection and analysis, internal and external validity, reporting, and applicability.
- *Experimental studies*: seven items covering study design, control group, random allocation, data collection and analysis, validity, and applicability.

Each item was scored 0 (not met) or 1 (met), and total scores were calculated. Studies meeting the defined quality thresholds were included in the final analysis. Quality assessment was performed independently by two researchers (ZKHF and MZ), with discrepancies resolved through group discussion to ensure uniform application of criteria and comparability of results (Table 2).

### Data analysis and synthesis

Data analysis and synthesis followed an integrative review approach according to Whittemore & Knafl (2005), allowing qualitative, quantitative, and mixed-methods studies to be analyzed and synthesized together [26].

1. Qualitative Studies:
  Data were analyzed using inductive content analysis and constant comparison as per Elo & Kyngäs (2008) (27).
  Initial codes were extracted and grouped into axial categories.
  Main themes and subthemes were identified.
2. Quantitative and Mixed-Methods Studies:
  Data were analyzed descriptively and narratively following Sandelowski, Voils & Barroso (2006) (28).
  Each result related to ethical dimensions of palliative care was labeled for integration with qualitative data.
3. Data Integration and Identification of Themes, Categories, and Domains:
  The analytical process followed a clear hierarchical structure to organize the findings. The most granular level of analysis resulted in specific themes, which represent concrete ethical issues or experiences. Related themes were then clustered into broader, more abstract categories. Finally, these categories were synthesized into the highest level of abstraction, the overarching domains. This three-level hierarchy (Themes → Categories → Domains) structures the presentation of results in Table 4 and guides the discussion.
  Data harmonization: Codes and findings from quantitative and mixed-methods studies were compared with qualitative themes, and similar items were grouped into preliminary themes.
  Category development: Harmonized themes were organized into broader categories reflecting various ethical challenges faced by nurses.
  Domain development: Categories were synthesized into main domains, including:
  1. Clinical decision-making and patient autonomy
  2. Justice and resource allocation
  3. Beneficence and patient-centered care
  4. Non-maleficence and prevention of moral distress
    Validation and Consensus: Quantitative findings were first labeled according to relevant ethical dimensions and then harmonized with qualitative themes. Two researchers independently reviewed codes and labels at each stage, and disagreements were resolved through discussion and consensus. An example of the comparison and harmonization process is presented in Additional File 2 (26, 29).
4. Final Reporting of Findings:
  Final results were presented in Table 2 to ensure transparency, reproducibility, and comparability.

**Table 2:** Quality assessment of included studies

Number of Studies	Study Type	Quality Assessment (Gifford et al., 2007)	Status
6	Systematic Review	High (met most criteria; clear methodology, transparent search and synthesis)	Acceptable
16	Qualitative	High (clear sampling, data collection, analysis; ethical considerations reported)	Acceptable
4	Quantitative / Cross-sectional	High (well-defined population, validated instruments, clear analysis)	Acceptable
2	Mixed Methods	High (integration of qualitative and quantitative data, transparent reporting)	Acceptable
3	Literature / Narrative Review	High (systematic or structured review process, coherent synthesis of evidence)	Acceptable
3	Survey / Early Reports	High (validated questionnaires, clear reporting of findings)	Acceptable

This stepwise and transparent approach ensures that themes and categories were systematically extracted from diverse studies and synthesized into final domains, enhancing the validity and reproducibility of the findings.

## Results

### Study selection process

A total of 1,201 records were identified through database searches, and an additional 12 records were found through website and grey literature searches. After removing 487 duplicates, 671 records were screened based on titles and abstracts. Full texts of 89 articles were assessed for eligibility, and ultimately 34 articles met the inclusion criteria (Table 3).

**Table 3:** General characteristics of studies included in the review

Author	Aim	Study Type	Country	Method	Sample Size	Domain	Category	Theme
Adegbesan et al. (2024) [1]	Explore ethical challenges in AI integration in palliative care	Concept analysis, review	International	Conceptual analysis	N/A	Beneficence & patient-centred care	Informed consent & data privacy	Innovations & emerging technologies
Akbarian-Rakeni et al. (2023) [2]	Investigate nurses’ perceptions of challenges in end-of-life care	Qualitative	Iran	Structured interviews	16 nurses	Clinical decision-making & patient autonomy	Conflict in clinical decision-making	Decision-making about treatment withdrawal
Alanazi et al. (2024) [3]	Review barriers to palliative care access in older adults	Systematic review	International	Systematic review	28 studies	Justice & resource allocation	Team communication & coordination	Lack of joint decision-making guidelines
Alodhialah et al. (2025) [4]	Explore palliative care access among older adults	Qualitative	Saudi Arabia	Interviews	22 nurses	Justice & resource allocation	Lack of education & organizational support	Pressure from cultural and religious differences
Arab et al. (2022) [5]	Investigate nurses’ experiences of ethical values in home care	Qualitative	Iran	Interviews, content analysis	16 nurses	Clinical decision-making & patient autonomy	Maintaining patient autonomy & dignity	Conflict between patient wishes and family expectations / Pressure from cultural and religious differences
Arash et al. (2024) [6]	Examine clinical decision-making and moral distress among ICU nurses	Descriptive-analytical, cross-sectional	Iran	Survey	198 nurses	Clinical decision-making & patient autonomy / Non-maleficence & moral distress prevention	Conflict in clinical decision-making / Psychological consequences for nurses	Continuing, withholding or limiting invasive treatment / Moral distress & ethical injury
Arends et al. (2022) [7]	Explore moral distress in life-prolonging treatments	Mixed-methods	Germany	Questionnaire & data analysis	15 nurses	Non-maleficence & moral distress prevention / Clinical decision-making & patient autonomy	Psychological consequences for nurses / Conflict in clinical decision-making	Moral distress & ethical injury / Continuing, withholding or limiting invasive treatment
Bosch et al. (2023) [8]	Examine ethical challenges among nurses and volunteers	Qualitative	Netherlands	Hermeneutic interviews	21 nurses	Clinical decision-making & patient autonomy / Non-maleficence & moral distress prevention	Maintaining patient autonomy & dignity / Psychological consequences for nurses	Conflict between patient wishes and family expectations / Pressure from cultural differences / Moral distress & ethical injury
Cheon et al. (2015) [9]	Ethical issues experienced by hospice & palliative nurses	Survey	USA	Questionnaire	861 nurses	Clinical decision-making & patient autonomy	Conflict in clinical decision-making	Forced continuation of non-beneficial care
Friedrichsen et al. (2024) [10]	Explore ethical challenges in thirst management for dying patients	Qualitative	Sweden	Semi-structured interviews, thematic analysis	14 nurses	Clinical decision-making & patient autonomy	Conflict in clinical decision-making	Medication & fluid management
Geng et al. (2024) [11]	Systematic review of ethical dilemmas for palliative care nurses	Systematic review	International	Systematic review	15 studies	Justice & resource allocation / Non-maleficence & moral distress prevention	Team communication & coordination / Psychological consequences for nurses	Communication problems / Moral distress & ethical injury
Ghavi et al. (2024) [12]	Ethical challenges & proposed strategies	Qualitative	Iran	Structured interviews	12 nurses	Non-maleficence & moral distress prevention / Justice & resource allocation	Psychological consequences for nurses / Lack of education & organizational support	Moral distress & ethical injury / Burnout / Workload & lack of support
Gonzalez-Perez et al. (2025) [13]	Ethical conflict perspectives	Qualitative	Spain	Semi-structured interviews	12 nurses	Justice & resource allocation	Lack of education & organizational support	Lack of professional ethics training
Geuenich et al. (2025) [14]	Investigate supervision, moral distress and injury in palliative care	Qualitative	Germany	Interviews & thematic analysis	20 nurses	Non-maleficence & moral distress prevention	Psychological consequences for nurses	Moral distress & ethical injury
Heggestad et al. (2021) [15]	Systematic review of ethical challenges in home-based care	Systematic review	Scandinavia	Systematic review	29 studies	Clinical decision-making & patient autonomy / Justice & resource allocation	Maintaining patient autonomy & dignity / Lack of education & organizational support	Conflict between patient wishes and family expectations / Workload pressure & lack of institutional support
Heshmatifar et al. (2024) [16]	Integrated review of team working barriers in elderly palliative care	Review	International	Integrated review	28 studies	Justice & resource allocation	Lack of education & organizational support / Team communication & coordination	Lack of professional ethics education / Workload / Lack of interprofessional collaboration
Ibrahim et al. (2024) [17]	Identify ethical issues and impact on nurse quality of life	Field study	Egypt	Survey	85 nurses	Clinical decision-making & patient autonomy	Conflict in clinical decision-making	Medication & fluid management
İlhan et al. (2024) [18]	Explore difficulties of palliative care nurses in emergency care	Qualitative	Turkey	Semi-structured interviews	12 nurses	Beneficence & patient-centred care / Justice & resource allocation	Care environment / Lack of education & organizational support	Emergency conditions & time pressure / Workload & lack of support
Kochems et al. (2023) [19]	Perceived barriers in providing palliative care in primary care & nursing homes	Cross-sectional	Netherlands	Questionnaire	84 nurses	Justice & resource allocation	Team communication & coordination / Lack of education & organizational support	Lack of interprofessional collaboration / Limited resources & time / Workload & lack of support
Luk & Chan (2018) [20]	Examine EOL care for advanced dementia patients	Qualitative	Hong Kong	Interviews	10 nurses	Non-maleficence & moral distress prevention	Legal & ethical challenges / Conflict in clinical decision-making	Fear of legal responsibility / Treatment decisions
Mehr-ol-Hosseni et al. (2021) [21]	Systematic review of palliative care challenges	Systematic review	Iran	Systematic literature search	N/A	Clinical decision-making & patient autonomy / Justice & resource allocation	Conflict in clinical decision-making / Lack of education & organizational support	Continuing, withholding or limiting invasive treatment / Lack of professional ethics training
Moazam et al. (2020) [22]	Review of ethical challenges in EOL care	Literature review	Iran	Text analysis	N/A	Clinical decision-making & patient autonomy	Conflict in clinical decision-making	Continuing, withholding or limiting invasive treatment
Muldrew et al. (2019) [23]	Mixed-method study on ethical issues in nursing homes	Qualitative & quantitative	UK	Interviews & questionnaire	40 nurses	Justice & resource allocation / Non-maleficence & moral distress prevention	Lack of education & organizational support / Psychological consequences for nurses	Workload & lack of institutional support / Lack of training / Moral distress & ethical injury
Muldrew et al. (2020) [24]	Survey on ethical issues in nursing home palliative care	Cross-sectional	UK & Canada	Questionnaire	469 nurses	Clinical decision-making & patient autonomy / Justice & resource allocation	Maintaining patient autonomy & dignity / Team communication	Respect for autonomy / Family stress management / Poor communication
Nikbakht et al. (2021) [25]	Phenomenological study of nurses’ moral distress in long-term care	Qualitative	Iran	Interviews	9 nurses	Justice & resource allocation / Non-maleficence & moral distress prevention	Lack of education & organizational support / Psychological consequences for nurses	Workload & lack of support
Omidi et al. (2025) [26]	Explore ICU nurses’ perceptions of palliative care	Qualitative	Iran	Phenomenology	16 nurses	Beneficence & patient-centred care / Clinical decision-making & patient autonomy	Care environment / Conflict in clinical decision-making	ICU care & treatment conflicts / Aggressive vs. palliative treatment
Peng et al. (2025) [27]	Examine moral distress, attitude toward death & palliative competencies	Cross-sectional	China	Questionnaire	342 nurses	Non-maleficence & moral distress prevention	Psychological consequences for nurses	Moral distress & ethical injury
Sadeghi et al. (2021) [28]	Nurses’ experiences of neonatal ICU palliative care	Qualitative	Iran	Semi-structured interviews	10 nurses	Clinical decision-making & patient autonomy	Conflict in clinical decision-making / Maintaining patient autonomy & dignity	Continuing, withholding or limiting invasive treatment / Conflict between patient wishes and care team
Schofield et al. (2021) [29]	Systematic review of ethical challenges in palliative care	Systematic review	International	Systematic review & thematic synthesis	43 studies	Clinical decision-making & patient autonomy	Conflict in clinical decision-making / Maintaining patient autonomy & dignity	Continuing, withholding or limiting invasive treatment / Use of palliative sedation / Conflict between patient wishes & family expectations
Svendsen et al. (2025) [30]	Nurses’ ethical compass in home-based palliative care	Qualitative	Norway	Thematic analysis	16 nurses	Clinical decision-making & patient autonomy	Maintaining patient autonomy & dignity	Respect for autonomy
Udeh et al. (2024) [31]	Qualitative study of ethical issues in EOL care	Qualitative	Nigeria	Thematic interviews	15 nurses	Non-maleficence & moral distress prevention / Clinical decision-making & patient autonomy	Legal & ethical challenges / Maintaining patient autonomy & dignity	Fear of legal responsibility / Conflict between patient and team expectations
Willmott et al. (2020) [32]	Nurses’ knowledge of law at EOL & implications	Qualitative	Australia	Semi-structured interviews	42 nurses	Non-maleficence & moral distress prevention	Legal & ethical challenges	Fear of legal responsibility
Wong et al. (2020) [33]	Quality of palliative care in Hong Kong	Cross-sectional	Hong Kong	Questionnaire	305 nurses	Non-maleficence & moral distress prevention	Legal & ethical challenges / Conflict in clinical decision-making	Fear of legal responsibility / Treatment decisions
Yildirim et al. (2025) [34]	Moral courage of palliative care nurses	Descriptive	Turkey	Questionnaire	121 nurses	Non-maleficence & moral distress prevention	Psychological consequences for nurses	Reduced moral courage

### Overall characteristics of included studies

All included studies focused on nurses (registered nurses, nursing assistants, or nursing staff) providing palliative or end-of-life care. The 34 studies were independent and published between 2015 and 2025, available in English or Persian. Eight studies were conducted in Iran, and the remainder were from other countries or international contexts.

Seventeen studies were qualitative, primarily using semi-structured or structured interviews with thematic or content analysis. Seven studies were quantitative, mostly cross-sectional using questionnaires, and seven were mixed-methods (interview + questionnaire or descriptive + analytical). The methodological quality of all studies was deemed acceptable based on Gifford et al.’s (2007) checklist [25].

Participants were predominantly hospital, home care, ICU, or hospice nurses, mostly female, with 1–25 years of work experience.

### Ethical challenges identified (Table 4)

1. Clinical Decision-Making and Patient Autonomy
  ◦Category 1.1: Conflicts in Clinical Decisions
    Theme: Continuation, withdrawal, or limitation of aggressive treatmentsNurses faced conflicts regarding continuation, withdrawal, or limitation of aggressive treatments, causing psychological stress and job strain [11, 19, 30].
    Theme: Medication and fluid management Decision-making in medication and fluid management often lacked clear guidelines, requiring balancing patient comfort with professional standards [11, 17].
    Theme: Use of palliative sedation Use of palliative sedation was challenging and required team coordination and adherence to professional ethics [10, 31].
  ◦Category 1.2: Maintaining Patient Autonomy and Dignity
    Theme: Conflicts between patient wishes and family expectationsNurses attempted to preserve patient dignity amidst conflicts between patient and family expectations [9, 12, 32].
    Theme: Cultural and religious differencesCultural and religious differences created ethical pressure in both home and hospital settings [9, 33, 34].
2. Justice and Resource Allocation
  ◦Category 2.1: Lack of Training and Organizational Support
    Theme: Inadequate specialized ethics trainingLack of specialized ethics education made ethical decision-making difficult and increased moral distress [35, 31, 30].
    Theme: High workload and lack of institutional supportHigh workload and insufficient institutional support contributed to burnout and reduced moral courage [36, 38].
  ◦Category 2.2: Communication and Team Coordination Issues
    Theme: Insufficient interdisciplinary collaborationLimited interdisciplinary collaboration led to incomplete decisions and heightened ethical conflicts [40, 39, 18].
    Theme: Resource and time constraintsScarce resources and time created ethical pressure and reduced quality of palliative care [17, 41].
    Theme: Absence of shared decision-making guidelinesLack of common clinical decision-making protocols led to more challenging and error-prone decisions [11, 18].
3. Beneficence and Patient-Centered Care
  ◦Category 3.1: Innovations and Emerging Technologies
    Theme: Use of artificial intelligence and ethical risksAI use in palliative care raised ethical concerns, including informed consent and data privacy [39].
  ◦Category 3.2: Care Environment and Setting
    Theme: Emergency situations and time pressureUrgent conditions and time constraints contributed to nurses’ moral distress [42, 36, 19, 12].
    Theme: ICU care and treatment conflictsICU nurses faced conflicts between aggressive treatment and palliative care approaches [43, 42, 36].
4. Non-Maleficence and Moral Distress Prevention
  ◦Category 4.1: Psychological Consequences for Nurses
    Theme: Moral distress and ethical injuryMoral distress and ethical injury were common among nurses [46–44, 42].
    Theme: BurnoutEthical and workload pressures led to occupational burnout [48, 47].
    Theme: Reduced moral courageInsufficient support and workload pressures reduced nurses’ moral courage [49].
  ◦Category 4.2: Legal and Regulatory Challenges
    Theme: Fear of legal responsibility and conflicts between patient and team preferences Ambiguity in end-of-life laws and concerns over legal consequences sometimes prevented proper clinical decisions and increased ethical stress, highlighting the need for organizational support [50, 42, 1].

**Table 4:** Hierarchy of nurses’ ethical challenges in palliative care: domains, categories, and themes

Domain	Category	Theme
1. Clinical decision-making & patient autonomy	1.1 Clinical decision-making conflicts	Continuation, withdrawal, or limitation of invasive treatments
		Medication and fluid management
		Use of palliative sedation
	1.2 Preserving patient autonomy and dignity	Conflict between patient wishes and family expectations
		Pressure arising from cultural and religious differences
2. Justice & resource allocation	2.1 Lack of education and organizational support	Lack of specialized professional ethics training
		Workload pressure and insufficient institutional support
	2.2 Communication and team coordination issues	Lack of interprofessional collaboration
		Limited resources and time
		Absence of shared decision-making guidelines
3. Beneficence & patient-centred care	3.1 Innovations and emerging technologies	Use of artificial intelligence and ethical risks
		Informed consent and data privacy
	3.2 Care environment and setting	Emergency conditions and time pressure
		ICU care and treatment-related conflicts
4. Non-maleficence & moral distress prevention	4.1 Psychological outcomes for nurses	Moral distress and moral injury
		Occupational burnout
		Reduced ethical courage
	4.2 Legal and regulatory challenges	Fear of legal liability
		Conflict between patient wishes and care team

### Barriers and facilitators of ethical care

Barriers and facilitators were mainly related to nurse-patient, nurse-team, and organizational interactions. Trust-based, respectful relationships, patient-centered approaches, and flexibility in care provision facilitated ethical care, while lack of knowledge, time pressure, and insufficient institutional support were major barriers [51, 12, 2].

### Practical recommendations

Recommendations included ethics training, promoting patient-centered care, enhancing interdisciplinary collaboration, improving organizational support, and resource allocation. Practical strategies involved scenario-based training, advance care planning, enhancing family communication, and better access to palliative care resources [52, 44].

## Discussion

This systematic review, drawing on 34 studies from hospital and home care settings, provides a multidimensional picture of the ethical challenges faced by nurses in delivering palliative care to patients with chronic illnesses. The findings address the three main research questions concerning nurses’ perspectives, ethical challenges, and practical strategies, and are organized into four overarching domains—clinical decision-making and patient autonomy, justice and resource allocation, beneficence and patient-centered care, and non-maleficence and the prevention of moral distress—supported by eight categories. Together, these domains form a coherent framework for understanding the complex nature of ethical issues in palliative care. 

In the domain of *clinical decision-making and patient autonomy*, the findings reveal that nurses frequently encounter situations involving decisions about continuing or withdrawing aggressive treatments, managing medications and fluids, or implementing palliative sedation. These scenarios, grouped under the category “clinical decision-making conflicts,” emerged as some of the most recurrent challenges because nurses often navigate tensions among their own clinical judgment, patient preferences, and family expectations [11, 17, 36, 43]. These dilemmas become even more complex in intensive care units and emergency settings, where rapid decisions carry significant ethical implications [19, 53]. Similarly, the category “preserving autonomy and patient dignity” illustrates how discrepancies between patient wishes and family demands—especially within strong cultural or religious contexts—play a critical role in shaping ethical decision-making [9, 12, 51]. These findings underscore that nurses’ ethical reasoning is shaped by a dynamic interplay of professional, cultural, and emotional factors.

The domain of *justice and resource allocation* highlights widespread agreement across studies that inadequate ethical training and insufficient organizational support are major obstacles to ethical practice. The category “lack of training and organizational support” shows that many nurses feel ill-equipped to handle ethical dilemmas due to insufficient training on end-of-life decision-making, ethical conflicts, and relevant legal frameworks [22, 31, 35, 38, 54]. This deficit contributes to uncertainty, hesitancy, and diminished professional confidence. Moreover, the category “communication and teamwork challenges” demonstrates how time constraints, limited interprofessional collaboration, and resource shortages hinder the implementation of ethical decisions and compromise justice in care delivery [14, 18, 30]. These results suggest that many ethical challenges arise not from individual shortcomings but from systemic organizational limitations.

The domain of *beneficence and patient-centered care* examines the influence of emerging technologies and workplace environments on ethical practice. Studies under the category “innovation and emerging technologies” indicate that the integration of AI-based tools into palliative care introduces new ethical concerns, including informed consent, data privacy, and technological bias [39]. Nurses must balance the benefits of technology in improving care quality with the potential ethical risks associated with its use. Furthermore, the category “care environment and clinical conditions” highlights how time pressure, high-acuity clinical settings, and intensive care environments affect not only ethical decision-making but also the feasibility of delivering patient-centered care [36, 42, 43]. These findings reveal that beneficence in palliative care is shaped not only by nurses’ competencies but also by structural and environmental conditions.

In the domain of *non-maleficence and the prevention of moral distress*, the psychological and legal consequences of ethical challenges take center stage. The category “psychological impacts on nurses” illustrates that repeated exposure to ethically challenging situations can lead to moral distress, moral injury, burnout, and reduced moral courage—outcomes consistently reported across diverse clinical settings (13, 32, 37, 45, 47, 54). Parallel to these concerns, the category “legal and regulatory challenges” shows that ambiguous end-of-life legislation, fear of legal consequences, and concerns about litigation may lead to excessive caution and hinder appropriate clinical decision-making [15, 49, 50]. These findings emphasize the importance of clear legal frameworks and supportive institutional policies.

From a cross-cultural perspective, the analysis of 34 studies indicates that although many countries experience similar ethical challenges, the intensity and nature of these challenges vary according to cultural norms, legal systems, and health-care infrastructures. In Middle Eastern countries, family involvement and cultural expectations play a significant role in shaping decision-making conflicts, whereas in European and Australian contexts, documentation standards and legal implications are more prominent [36, 55]. These differences highlight the need for culturally sensitive ethical interventions tailored to specific care environments.

Several *knowledge gaps* emerged from the review. First, few studies evaluated the effectiveness of educational or organizational interventions for reducing moral distress, despite widespread acknowledgment of their necessity. Second, the evidence base remains disproportionately focused on hospital settings, with fewer studies examining ethical challenges in home-based palliative care. Third, limited research has explored the ethical implications of emerging technologies, despite their increasing integration into palliative care. Lastly, although many studies referenced legal and policy influences, few examined their practical impact on ethical decision-making through empirical methods.

Beyond identifying challenges, this review synthesizes a range of *practical strategies* proposed across the included studies, aligning with the third research question. These include structured ethics education, development of shared decision-making guidelines, strengthening interprofessional collaboration, establishing support systems to reduce moral distress, and adopting culturally sensitive policies. Additionally, revising end-of-life and professional responsibility legislation may enhance nurses’ confidence and reduce fear-driven or overly cautious decision-making.

Overall, the findings of this review demonstrate that nurses’ ethical challenges arise from a complex interaction among ethical knowledge, organizational structures, cultural influences, legal constraints, and psychological consequences. A key strength of this review lies in its integration of evidence across diverse settings and countries, providing a comprehensive, multilayered understanding of the ethical landscape in palliative care. These insights can inform the development of educational programs, organizational interventions, and policy reforms aimed at strengthening ethical palliative care practice.

### Strengths and limitations

One strength of this systematic review is its focus on nurses’ reported ethical challenges, moral distress, and care experiences in palliative care for patients with chronic illnesses. By systematically identifying and synthesizing themes, categories, and domains, the review provides a structured overview of ethical issues. Broad inclusion criteria allowed for a substantial number of studies encompassing diverse nursing perspectives. Moreover, including qualitative, quantitative, and mixed-methods studies facilitated the integration of varied viewpoints across care settings.

Limitations include variability in the definitions and terminology of palliative and end-of-life care across studies, which may have led to inclusion of research examining related but distinct aspects of care. Some qualitative findings are based on participants’ perspectives and experiences rather than strictly empirical data. Only studies published in Persian and English were included, possibly overlooking relevant research in other languages. Additionally, a few studies did not report sufficient methodological details, which may have underestimated their rigor.

## Conclusion

The findings of this systematic review indicate that nurses providing palliative care to patients with chronic illnesses encounter multiple ethical challenges that may influence care quality and their psychological well-being. Challenges include clinical decision-making, maintaining patient dignity and autonomy, lack of organizational support and training, communication and team-related issues, psychological stress, legal and regulatory concerns, and the influence of care environments and technology.

Organizational support, ongoing professional ethics education, and clear frameworks for clinical decision-making may help reduce moral distress and occupational burnout. Strengthening communication skills among team members and with patients and families, considering cultural and religious differences, and providing practical guidance in emergency situations are also important.

Future research should focus on the development and evaluation of interventions to operationalize ethical frameworks and support nurses across diverse palliative care settings. Investigating the effectiveness of ethics education, strategies to reduce moral distress, and approaches to improve interprofessional collaboration may further enhance both care quality and nurses’ well-being.

## Supplementary Information

Supplementary Material 1.

Supplementary Material 2.

## Data Availability

All data analyzed during this study are derived from published sources and are available in the cited references.

## Abbreviations

PRISMA: Preferred reporting items for systematic reviews and meta-analysis
WHO: World Health Organization
