# Supplementary material for: Ethical perspectives in palliative care for chronic patients: a systematic review of nurses’ experiences in home and hospital settings
Source: BMC Palliat Care. 2026 Mar 12;25:104. doi: 10.1186/s12904-026-02032-0 (PMC13097765; doi:10.1186/s12904-026-02032-0)
Supplement: Supplementary file 1 — Supplementary Material 1. [file 12904_2026_2032_MOESM1_ESM.docx]

**Sample of Data Comparison and Alignment Process**

| **Study ID** | **Quantitative Finding** | **Qualitative Theme** | **Aligned Category** | **Domain** |
| --- | --- | --- | --- | --- |
| Smith et al., 2020 | 42% of nurses reported moral distress when administering end-of-life care against patient wishes | Ethical conflict in clinical decision-making | Clinical decision-making and patient autonomy | Decision-making & Patient Autonomy |
| Li & Chen, 2019 | High workload and insufficient resources negatively affected ethical practice | Resource constraints and moral stress | Justice and resource allocation | Justice & Resource Access |
| Akbarian-Rokni et al., 2023 | 65% of nurses struggled with informed consent for palliative interventions | Maintaining patient autonomy and privacy | Beneficence and patient-centered care | Beneficence & Patient-Centered Care |
| Friedrichsen et al., 2024 | Moral distress reported due to conflicting family and patient preferences | Moral distress and burnout | Non-maleficence and moral distress prevention | Non-Maleficence & Moral Distress Prevention |

**Notes on the process:**

1. Quantitative results were labeled according to ethical dimensions.
2. Qualitative themes were extracted via inductive content analysis.
3. Quantitative findings were systematically matched to corresponding qualitative themes to form unified categories.
4. Categories were then grouped into overarching domains reflecting major ethical challenges.
5. Two independent researchers reviewed all codes and alignments; discrepancies were resolved through discussion and consensus.
