# Supplementary material for: Ethical perspectives in palliative care for chronic patients: a systematic review of nurses’ experiences in home and hospital settings
Source: BMC Palliat Care. 2026 Mar 12;25:104. doi: 10.1186/s12904-026-02032-0 (PMC13097765; doi:10.1186/s12904-026-02032-0)
Supplement: Supplementary file 2 — Supplementary Material 2. [file 12904_2026_2032_MOESM2_ESM.docx]

**Additional file 1. Search Strategies Used in All Databases**

**1. PubMed Search Strategy**

("Palliative Care"[Mesh] OR "Hospice Care"[Mesh] OR palliative care[tiab] OR end-of-life care[tiab]

OR hospice[tiab] OR supportive care[tiab])

AND

(nursing[tiab] OR nurse*[tiab] OR "Nursing"[Mesh])

AND

(ethical challenge*[tiab] OR ethical dilemma*[tiab] OR moral distress[tiab]

OR "Ethics, Nursing"[Mesh] OR ethical issue*[tiab] OR ethical decision-making[tiab])

AND

(chronic disease[tiab] OR chronic illness[tiab] OR "Chronic Disease"[Mesh]

OR life-limiting illness[tiab] OR serious illness[tiab])

AND

("2015/01/01"[Date - Publication] : "2025/04/30"[Date - Publication])

**2. Scopus Search Strategy**

(TITLE-ABS-KEY("palliative care" OR "end-of-life care" OR "hospice" OR "supportive care"))

AND

(TITLE-ABS-KEY(nurse* OR nursing))

AND

(TITLE-ABS-KEY("ethical challenge*" OR "ethical dilemma*" OR "moral distress"

OR "ethical issue*" OR "ethical decision making"))

AND

(TITLE-ABS-KEY("chronic disease" OR "chronic illness" OR "life-limiting illness" OR "serious illness"))

AND

(PUBYEAR > 2015 AND PUBYEAR < 2025)

**3. Web of Science Search Strategy**

TS=("palliative care" OR "end-of-life care" OR hospice OR "supportive care")

AND

TS=(nurse* OR nursing)

AND

TS=("ethical challenge*" OR "ethical dilemma*" OR "moral distress"

OR "ethical issue*" OR "ethical decision making")

AND

TS=("chronic disease" OR "chronic illness" OR "life-limiting illness" OR "serious illness")

Refined by: DOCUMENT TYPES = (Article OR Review)

Timespan: 2015–2025

**4. CINAHL (EBSCO) Search Strategy**

(palliative care OR end-of-life care OR hospice OR supportive care)

AND

(nurse* OR nursing)

AND

(ethical challenge* OR ethical dilemma* OR moral distress OR ethical issue* OR ethical decision making)

AND

(chronic disease OR chronic illness OR life-limiting illness OR serious illness)

Limiters:

• Published Date: 20150101–20250430

• English OR Persian

• Peer-reviewed

**5. Google Scholar (for grey literature)**

Keywords used:

"palliative care" "nursing" "ethical challenges" "ethical dilemmas" "moral distress" chronic illness

Screening of the first 200 results was conducted, consistent with recommendations for grey literature searching.

**6. Persian Databases (SID, Magiran)**

"مراقبت تسکینی" OR "مراقبت پایان زندگی" OR "پرستاری"

AND

"چالش اخلاقی" OR "دوراهی اخلاقی" OR "پریشانی اخلاقی" OR "تصمیم‌گیری اخلاقی"

AND

"بیماری مزمن" OR "بیماری محدودکننده حیات"
